# Commentary: Oxytocin-gaze positive loop and the coevolution of human–dog bonds

**DOI:** 10.3389/fnins.2016.00155

**Published:** 2016-04-11

**Authors:** Zoltan Kekecs, Aba Szollosi, Bence Palfi, Barnabas Szaszi, Krisztina J. Kovacs, Zoltan Dienes, Balazs Aczel

**Affiliations:** ^1^Department of Psychology and Neuroscience, Baylor UniversityWaco, TX, USA; ^2^Department of Affective Psychology, Institute of Psychology, Eötvös Loránd UniversityBudapest, Hungary; ^3^Department of Affective Psychology, Doctoral School of Psychology, Eötvös Loránd UniversityBudapest, Hungary; ^4^Laboratory of Molecular Neuroendocrinology, Institute of Experimental MedicineBudapest, Hungary; ^5^School of Psychology, Sackler Centre for Consciousness Science, University of SussexBrighton, UK

**Keywords:** oxytocin, human–dog co-evolution, hand-reared wolves

It has been proposed that evolution of dogs have led to a set of changes, which made them functionally similar to humans in some cognitive, behavioral, and social aspects (Topál et al., 2005; MacLean and Hare, 2015). Searching for these similarities, Nagasawa et al. (2015) hypothesize an oxytocin-mediated positive loop, which developed through the coevolution of human–dog bonding. To test this hypothesis, they conducted a highly original experiment, examining the effects of a 30-min human–dog interaction on oxytocin-secretion in both owners and dogs, and investigating which characteristics of the interaction modulated the oxytocin change (experiment 1). A unique feature of the study is that the same experiment was repeated with hand-reared wolves and their owners to evaluate whether the proposed oxytocin loop was specific to the human–dog interaction. In a following experiment (experiment 2), they administered oxytocin to dogs, and recoded changes in social behavior, and effects of the behavioral change on the owner's urinary oxytocin levels.

In this commentary, we focus on experiment 1. In experiment 1, Nagasawa et al. (2015) found that oxytocin levels increased in dogs in the “long gaze group” (LG) and their owners, but not in wolves and their owners; and that dog-to-owner gaze predicted the oxytocin change in both dog owners and dogs, but not in wolf owners and wolves. They interpreted these findings as supporting the existence of a species-specific oxytocin reinforcement loop between humans and dogs. We show that the generalizability of these findings is questionable, and the proposed oxytocin positive loop acquired through the coevolution of dogs and humans is not yet justified.

First, an important limitation of the study is the presence of confounding differences between the dog and the wolf arm of the experiment. There are marked differences between males and females in the expression of and the response to oxytocin (Insel and Hulihan, 1995; Yamamoto et al., 2004; Ditzen et al., 2012; Rilling et al., 2014), which are mediated by sex steroids and behavioral factors (Ježová et al., 1996; Petersson et al., 1998; Uvnäs-Moberg, 2003). It has been found that after interaction with their bonded dogs, only women owners showed increased oxytocin levels, while oxytocin levels stayed the same or decreased in men (Miller et al., 2009). Nagasawa et al. (2015) acknowledge these results, and test for animal-sex effects, but they do not take into account owner-sex. Re-analysing their data, we found that oxytocin increase is evident in women owners of both dogs and wolves while oxytocin did not change or even showed a slight decrease in men. The sex effect on oxytocin change (female owner vs. male owner, χ^2^ = 3.69, *df* = 1, *p* = 0.054, proportion of pairs supporting the alternative hypothesis = 0.69) is in fact larger than the effect of the species (dog owners vs. wolf owners, χ^2^ = 0.19, *df* = 1, *p* = 0.662, proportion of pairs supporting the alternative hypothesis = 0.54). Thus, sex differences provide an alternative explanation for the finding that oxytocin increase was significantly higher in the owners of LG dogs compared to wolves. Dog owners were almost exclusively females (82% female), who are known to be highly responsive to interaction with their bonded pets, while average oxytocin change in wolf owners is decreased by the high percentage of males (55% female).

There were several other potentially confounding dissimilarities between the dog and the wolf arm of the experiment. Specifically, the rearing and socialization of animals was different. The authors show that there was no statistically significant effect of age at separation and duration of interaction on oxytocin change within wolves and acquisition age and socialization level within dogs. However, they examined different indicators of early-life experiences for the two species and did not do inter-species comparison. Additionally, there was a significant difference in the baseline oxytocin values of the dog and wolf owners (χ^2^ = 4.67, *df* = 1, *p* = 0.031). Thus, the apparent difference between dogs and wolf in inducing an oxytocin response in humans may be simply due to a ceiling effect.

Another problem lies in the inappropriate analysis methods applied for hypothesis testing. The authors found that interacting partner's oxytocin change and the duration of dog-to-owner gaze predicts the oxytocin change in dogs and their owners, but not significantly in wolves and their owners. However, these group-by-group analyses are not sufficient to test the authors' hypothesis that there are differences in the way that oxytocin change is influenced in dog and wolf owners. A more appropriate method would have been to build statistical models testing the effect of group-predictor interaction on oxytocin change. After building the appropriate models using the data of Nagasawa et al. we found no significant group-predictor interaction effects in owners or pets, leaving the authors' conclusions about the uniqueness of the human–dog oxytocin loop unsupported.

Finally, results are hard to interpret because of the extremely low statistical power. For example, the authors argue that the significant correlation between animal-to-owner gaze and oxytocin change found in dog owners but not wolf owners supports the conclusion that the oxytocin response to dogs' gaze is substantially different from that to wolves' gaze. This analysis is based on a sample of 28 dogs and 11 wolves. It is easy to demonstrate with bootstrap sampling from the dog dataset that even if the correlation between gazing and oxytocin change was as high in the wolf population as that found in the dog group, a sample of 11 wolves would not reveal statistically significant correlation in 71% of the samples. Assuming *r* = 0.53, correlation reported by Nagasawa et al. (2015) in the dog group and *r* = 0 in the wolf group, a statistical simulation using 10,000 simulated samples shows that a total sample of 88 (44 wolves) would be needed to detect an interaction effect with a power of 0.8 if we use a similar research design. For a group-by-group analysis, a Bayes factor (Dienes, 2014; Wagenmakers et al., 2015) on the *r* = 0 for wolves also indicates that 26 wolves would be necessary to obtain evidence against the correlation of wolf-to-owner gaze and oxytocin change in owners (Figure 1).

**Figure 1:**
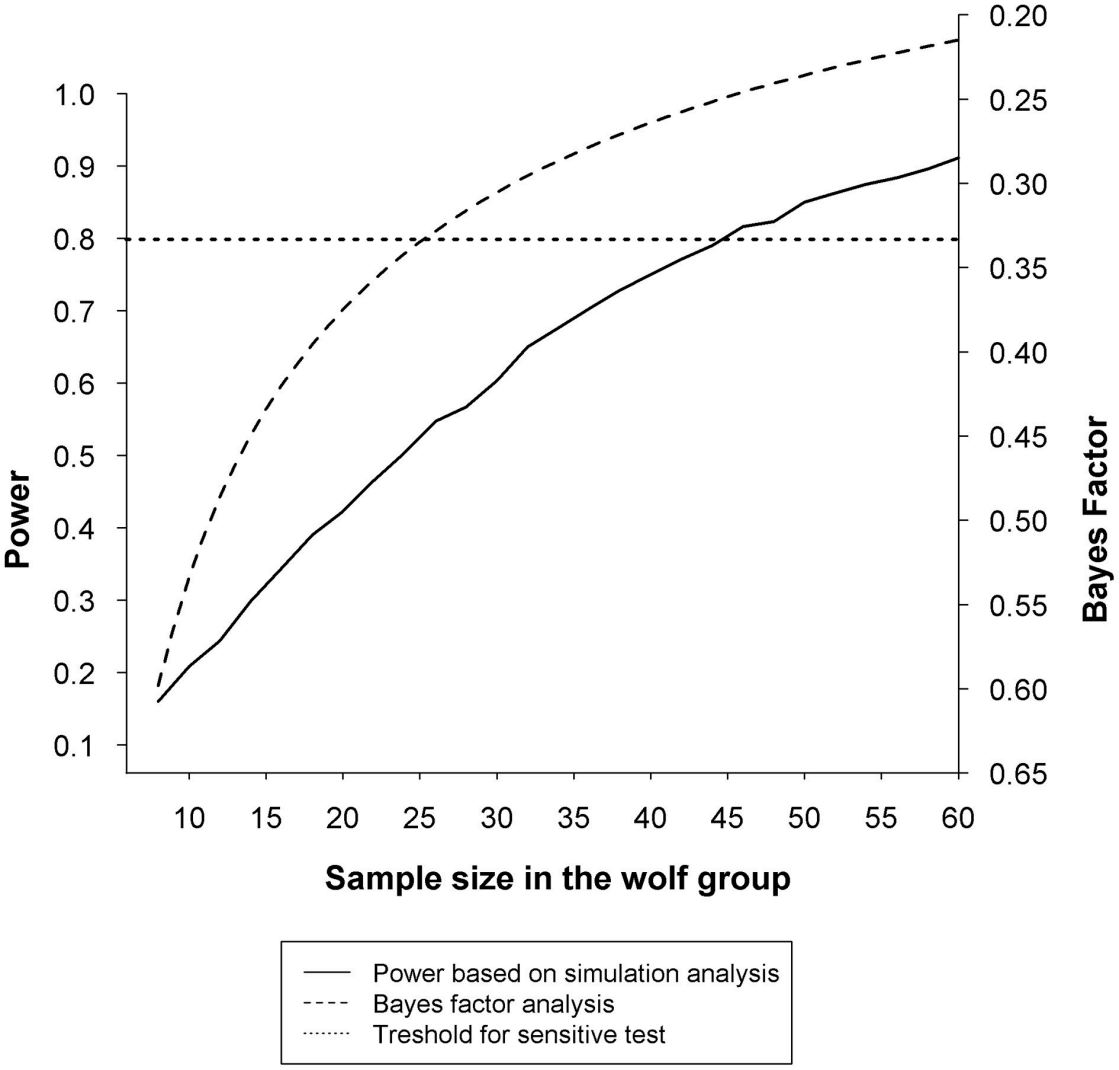
**Statistical sensitivity relative to sample size when testing the association between oxytocin change in owners and duration of animal-to-owner gaze**. The solid line represents the statistical power based on 10,000 simulated samples at each sample size of the wolf group between 8 and 60. Simulated samples were generated to match the observed correlation reported by Nagasawa et al. (2015) (*r* = 0.53) in the dog group and the theoretical correlation of *r* = 0 in the wolf group. Results show that 44 wolves (and the same number of dogs) would be necessary to show significant difference in the association between oxytocin change in owners and duration of animal-to-owner gaze in at least 80% of the samples (*power* = 0.8). The dashed line represents the Bayes Factor at each sample size between 8 and 60 calculated with a half-normal prior based on the observed correlation (*r* = 0.53) in the dog group and the theoretical correlation of *r* = 0 in the wolf group. A Bayes factor on the *r* = 0 for wolves indicates that due to the low sample size, the data are insensitive and do not provide evidence for the null hypothesis [*B*_H(0, Fisher'*s z*(0.60))_ = 0.51; see (Dienes, 2014)], and that at least 26 wolves would be required for a sensitive test [*B*_H(0, Fisher'*s z*(0.60))_ = 0.33] to provide evidence against correlation between oxytocin change in owners and duration of animal-to-owner gaze. The dotted line marks the thresholds for statistical sensitivity regularly used in the literature: power = 0.8 and *B* = 0.33.

Directly increasing sample size might be problematic, because of the scarcity of wolves, thus, we suggest alternative methods to increase power, such as taking several samples from the same animal (and owner) at different time-points or at different experimental sessions and taking into account inter-individual correlation using mixed models (Wang and Goonewardene, 2004). Simulation of this repeated measures procedure (assuming 0.7 inter-correlation between repeated measurements) indicates that, if we were limited to work with 11 wolves and the same number of dogs, we would have to take four measurements from the same animal to achieve sufficient statistical power.

In summary, Nagasawa et al. (2015) uses a novel experimental approach to examine the effects of human–dog interaction. Their experiment yields important knowledge regarding the existence and modulators of an oxytocin loop between animals and their owners, and the design also holds the potential to test whether this oxytocin loop is unique to the human–dog interaction. The importance and originality of this study is undeniable. However, we argue that methodological and statistical limitations of the current study prevent us from concluding that this loop was acquired during domestication of the dog. We recommend closer matching of the experimental arms (especially in sex distribution), more specific statistical hypothesis testing, and increasing statistical power by repeated measurements to test this hypothesis in future studies.

## Author contributions

ZK conceptualized the paper, drafted the manuscript, did the main data analyses and interpretation of the results, and handled correspondence with the authors of the commented paper. AS participated in the literature review for the manuscript, data analysis, and interpretation of the results as well as reviewed and edited the manuscript. He also helped in author correspondence. BP participated in the literature review for the manuscript, data analysis and interpretation of the results as well as reviewed and edited the manuscript. BS participated in the literature review for the manuscript. He also reviewed and edited the manuscript. KK helped to conceptualize the paper and reviewed and edited the manuscript. ZD helped in statistical considerations and reviewed and edited the manuscript. BA helped to conceptualize the paper, participated in the literature review for the manuscript. He also reviewed and edited the manuscript. All authors agreed to the final version of the manuscript.

### Conflict of interest statement

The authors declare that the research was conducted in the absence of any commercial or financial relationships that could be construed as a potential conflict of interest. The reviewer AM declared a shared affiliation with authors AS, BP, BS, and BA to the handling Editor, who ensured that the process met the standards of a fair and objective review.
